# Long-term outcomes and trends in elbow arthroplasty with Coonrad-Morrey prosthesis: a retrospective study in large group of patients

**DOI:** 10.1007/s00264-024-06272-8

**Published:** 2024-08-22

**Authors:** Luigi Tarallo, Andrea Celli, Matilde Delvecchio, Lorenzo Costabile, Grazia Ciacca, Giuseppe Porcellini, Fabio Catani

**Affiliations:** 1 Department of Orthopaedics and Traumatology, University of Modena and Reggio Emilia, Policlinico di Modena, Modena, Italy; 2 Department of Orthopaedics and Traumatology, Hesperia Hospital, Modena, Italy

**Keywords:** Elbow, Fracture, Prosthesis, Elbow prosthesis complications, Elbow prosthesis survival

## Abstract

**Purpose:**

Total Elbow Arthroplasty (TEA) was first developed to treat severe rheumatoid arthritis, but its uses have grown to encompass end-stage osteoarthritis, post-traumatic arthritis, and distal humeral fractures. This study analyzes indications changes, long-term survival, complications, and post-operative functional results of the Coonrad-Morrey prostheses, enhancing the existing literature on this technique and substantial case history.

**Methods:**

We included 122 arthroplasties in 117 patients, 28 males and 89 females (mean age of 67 years) treated in our hospital between 2002 and 2016. Minimum follow-up was four years. We collect functional parameters of 48 patients (51 elbows), due to death of patients due to old age and loss at follow-up.

**Results:**

Survival rate at five years was 90%, 85% at 10 years and 83% at 15 years. The overall medium Mayo elbow score was 79.7 ± 18.3 with the highest result in osteoarthritis patients (*p* < 0.005); QuickDASH score was 33.1 ± 25.5 with the worse result in rheumatoid group. Average post-operative arc of motion (ROM) was 95°±27°. There were complications in 46 out of 122 cases (37.7%) and revision surgeries were performed in 12 of them (9.8%): seven aseptic loosening, four late septic loosening, one bushing wear. In 27 instances (22.1%) was reported ulnar nerve involvement.

**Conclusion:**

Coonrad-Morrey prosthesis has shown satisfactory clinical results in the treatment of a wide range of pathologies. The long-term implant survivorship was satisfactory, yet the occurrence of failures and complications cannot be overlooked, above all the ulnar nerve paresthesia. There was a good recovery in quality of life, pain-free with limited residual limb disability.

## Introduction

**Fig. 1:**
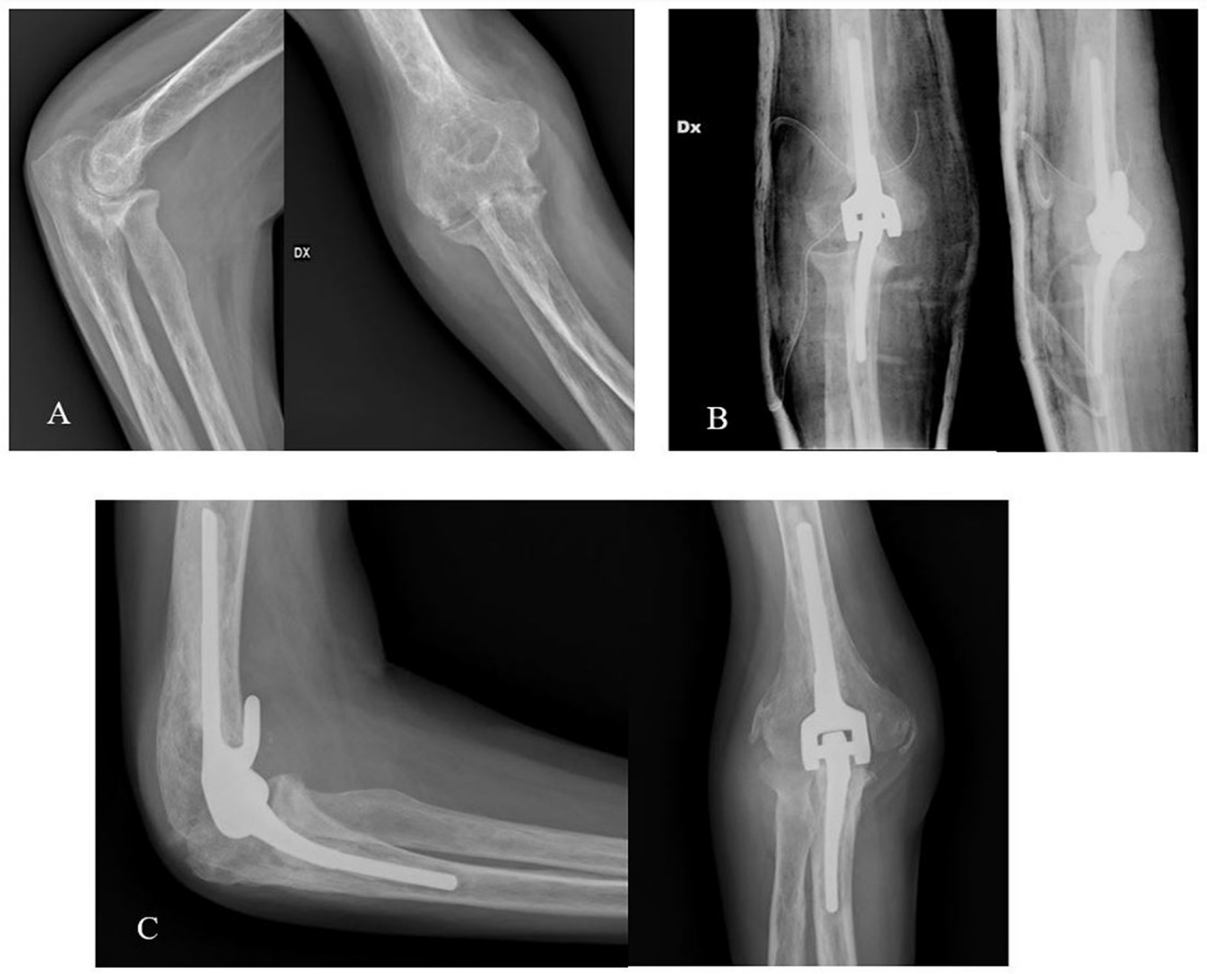
Sequence of radiographs from a clinical case of an elbow affected by primary osteoarthritis. (**A**) Preoperative x-rays; (**B**) Post-operative x-rays; (**C**) Follow-up x-rays one year after surgery

Total Elbow Arthroplasty (TEA) was first developed to treat severe rheumatoid arthritis. However, its uses have grown to encompass osteoarthritis, post-traumatic arthritis, irreversible distal humeral fractures, and unfavourable trauma sequelae [1]. Due to their highly limited design, early hinged implants failed by aseptic loosening of the humeral component. This mode of failure is caused by stress concentrations at the bone-cement interface [2]. Coonrad and Morrey made two significant inventions in the late 1970s: the anterior flange and the semiconstrained hinge. Although the humeral and ulnar components are mechanically linked to prevent instability, the semiconstrained hinge allows a modest degree of varus-valgus motion, which reduces stress concentration at the bone-cement interface [3]. When compared to a strictly intramedullary humeral component, the anterior flange addition to the humeral component also lessens rotational stress on the bone-cement contact [4] (Fig. 1). Consequently, despite the superior functional outcomes and survival observed in semiconstrained implants compared to unconstrained or fully constrained hinged implants [4], there is limited literature on long term Coonrad-Morrey prosthesis survivorship and clinical outcomes, especially with a substantial patient cohort.

The entire series of patients operated on by our Institute from January 2002 to September 2016 was therefore evaluated, with the aim of analyzing trends of indications regarding the use of the Coonrad-Morrey elbow arthroplasty, how these indications have changed over time, prosthesis survival, and complication incidence.

Another goal is the evaluation of the post-operative functional results and the analysis of the long-term survival of the Coonrad-Morrey implant, trying to compare any differences in terms of results due to the indication that led to the surgery.

## Materials and methods

A retrospective study was performed on patients who underwent primary elbow arthroplasty surgery with Coonrad-Morrey (CM) type prosthesis at our Orthopedic Department between 2002 and 2016 with a minimal follow-up of four years. The causes that led to prosthetic elbow replacement were recorded: rheumatoid arthritis (RA), ankylosis (ANK), severe instability of the elbow not otherwise approachable with less-invasive surgeries (INS), primary or post-traumatic osteoarthritis (OA), and non-synthesizable fractures of the distal humerus (FX).

All patients who underwent elbow arthroplasty with a Coonrad III type implant (known as “Coonrad-Morrey”, CM) were included in the study, excluding prosthetic revisions and patients who underwent a primary implant with CM prosthesis with causes not clearly attributable to the aforementioned diagnostic categories proposed.

All patients underwent surgery by two experienced surgeons in shoulder and elbow surgery, through triceps sparing approach (Alonso-Llames), to reduce the rate of complications in the extensor apparatus [5, 6]. 

During the selected time frame (168 months), 117 patients were operated on for a total of 122 prostheses implanted (five patients underwent bilateral prosthetic replacement); the study involved 28 men and 89 women (24% and 76% respectively), with an average age of 67.75 ± 12.87 years (range between 28 and 91 years old). The diagnoses that led to the intervention were collected: 18 RA (14.7%), 17 ANK due to heterotopic ossification (13.9%), 18 INS (14.8%), 25 OA (20.5%) and 44 FX (36.1%). Of the five bilateral prosthetic replacements, two cases involved patients suffering from bilateral RA and one from bilateral OA; the remaining two patients were both affected by RA, but while the first prosthesis was placed electively, the second implant was placed acutely on a fracture.

Subsequently, two equivalent time periods in terms of duration were arbitrarily taken into consideration (84 months; interval A: from January 2002 until September 2009; interval B: from October 2009 until September 2016). Within these homogeneous time intervals we analyzed how indications have changed over the years, after the increase in the use of DMARDs in clinical practice.

In the first group (interval A) there are 76 cases of implants: 16 RA (21.1%), 18 FX (23.7%), 16 OA (21.1%), 15 ANK (19.7%) and 11 INS (14.5%); and in interval B we find 46 implants: two RA (4.3%), 26 FX (56.5%), nine OA (19.6%), three ANK (6.5%) and six INS (13.0%). There is a general decline in the total number of CM implants with a simultaneous increase for the acute treatment of fractures of the distal humerus.

Graphics 1 summarizes the distribution of the prostheses implanted in the time frames considered.

During the follow-up assessment, two clinical questionnaires were used to evaluate the functionality of the joint: MEPS (Mayo Elbow Performance Score) which evaluates the functionality of the elbow joint by assigning a score, with a value from 0 to 100 [7–9] and the QuickDASH [10, 11] or the simplified version of the DASH score (“Disabilities of the Arm, Shoulder, and Hand”) [12], a non-specific test for the elbow that evaluates the residual disability of the upper limb.

Iatrogenic lesions affecting the ulnar nerve were evaluated with McGowan–Golberg classification based on symptoms and/or clinician opinion, without objective measures of function. It’s an increasing score as the patient’s clinical manifestations worsened: grade I sensory symptoms alone (positive Tinel sign), grade II sensory symptoms and mild muscle weakness (Froment’s sign), grade III sensory symptoms and severe muscle weakness or paralysis (hypotrophy of the hypothenar eminence and interosseous muscles, claw hand or total interruption of ulnar nerve conduction) [13]. The range of motion, expressed in degrees, is evaluated using a Biometrics Inclinometer instrument. Pain evaluation was conducted utilizing the Visual Analogue Scale (VAS) [14]. Finally, patient’s subjective level of satisfaction was assessed regarding the obtained result, based on the patient’s resumption of daily activities and their autonomy, using a score ranging from one (very dissatisfied) to five (fully satisfied).

### Statistical analysis

The data collected during the clinical follow-up of was subjected to univariate linear regression. Survival analysis was conducted using the Kaplan-Meier method and a Cox regression. A p–value ≤ 0.05 was considered statistically significant. Lastly, the statistical analysis was conducted using STATA 14.2.

## Results

### Clinical evaluation

Through the clinical re-evaluation of patients started in May 2021 we managed to collect the functional parameters of 48 patients (51 elbows) with an average age of 67 ± 10.4 years (range from 28 to 91 years) and an average follow-up of 9.9 years (range from 4.6 to 17.5 years). According to the MEPS questionnaire, 21 patients (41.2%) obtained a considerable “excellent” score (greater than 90 points), 14 (27.4%) a “good” result (between 75 and 89), nine (17 0.6%) a “sufficient” value (between 60 and 74), while seven (13.7%) obtained a “poor” score (less than 60). The average score was 79.7 ± 18.3 and 68.6% of patients achieved a rating between good and excellent. Upon evaluation the average ROM was 95°±27° with 33 elbows (64.7%) that presented an ROM greater than 100°, 13 (25.4%) between 100° and 50° and only five (9.8%) less than 50°. The post-operative residual disability of the operated upper limb was investigated using the QuickDASH questionnaire: the average score obtained was 33.1 ± 25.5.

Pain assessment was conducted using the VAS scale; the average result was 1.2 out of ten, with 37 out of 51 elbows (72.5%) being pain-free (0 out of 10).

Patient overall satisfaction with the obtained result from the surgical procedure was assessed and the mean score is 4.0 ± 1.0.

A clinical assessment was performed to evaluate any lingering impairment in nerve conduction attributed to the ulnar nerve, ranging from only sensitive symptoms and positive Tinel sign (grade I) in 14 patients to more severe manifestations like Froment sign (grade II) in six patients or hypotrophy of the hypothenar eminence (grade III) in sevent cases; the evaluation led to an average value of 0.9 (SD = 1.1). The results obtained were further divided and analyzed in distinct cohorts of patients based on the initial pathology leading to the elbow prosthesis implantation: seven RA (13.7%), 18 FX (35.3%), 11 OA (21.6%), eight ANK (15.7%) and seven INS (13.7%). The results are summarized in Table 1.

**Table 1:** Results of patient reassessment starting in May 2021; average monitoring of 9.9 years

Total *N*° of cases: 51	RA	FX	OA	ANK	INS
Number of patients	7	18	11	8	7
Range of motion	82,1 ± 33,6	96,1 ± 25,9	108,1 ± 13,3	84,4 ± 36,6	100 ± 23,1
MEPS	75 ± 26,0	81 ± 14,9	83,6 ± 14,3	74,4 ± 24,6	80,7 ± 19,0
QuickDASH	46,4 ± 37,1	30,1 21,2	36,1 ± 32,2	25,75 ± 21,1	31,4 ± 19,8
VAS	1,1 ± 2,0	0,7 ± 1,4	0,9 ± 1,4	1,5 ± 2,1	0,6 ± 1,5
Ulnar nerve disorders	1,4 ± 1,3	1,1 ± 1,1	0,9 ± 1,2	0,6 ± 0,7	0,4 ± 0,5
Satisfaction rate	3,7 ± 1,3	4,1 ± 1,0	4,0 ± 0,9	3,6 ± 1,4	4,1 ± 0,9
Age (mean)	60,7 ± 7,0	74,4 ± 8,1	65,4 ± 9,8	58 ± 8,5	66 ± 10,0
Average monitoring	148 ± 47,1	84,6 ± 45,7	130,5 ± 48,5	129,8 ± 33,7	146,6 ± 39,8

Regarding joint mobility, the best results were achieved by the cohort of patients with OA (average ROM 108°), while the worst outcomes pertain to patients in the ANK and RA groups (average ROM 84° and 82° respectively). The OA group’s best result for this parameter showed statistical significance (*p* < 0.005).

The MEPS questionnaire, similar to the mobility assessment, revealed best outcomes in patients with OA (mean MEPS score: OA 83.6; FX 81.1; INS 80.7; RA 75; ANK 74.4) without any statistical significance when compared with other groups (p-value > 0,05) (Table 2).

**Table 2:** Results of statistical analysis of scores and ulnar nerve disease

Variable	Comparison		*p*-value
ROM	OA	ANK	0,222
ROM	OA	RA	**0**,**037**
ROM	OA	INS	0,744
ROM	OA	FX	0,336
MEPS score	OA	ANK	0,586
MEPS score	OA	RA	0,680
MEPS score	OA	INS	0,781
MEPS score	OA	FX	0,682
QUICK DASH score	RA	ANK	0,728
QUICK DASH score	RA	OA	0,525
QUICK DASH score	RA	INS	0,949
QUICK DASH score	RA	FX	0,628
Ulnar nerve disorder	RA	ANK	0,220
Ulnar nerve disorder	RA	OA	0,675
Ulnar nerve disorder	RA	INS	0,074
Ulnar nerve disorder	RA	FX	0,283
Satisfaction	FX	ANK	0,464
Satisfaction	FX	RA	0,718
Satisfaction	FX	INS	0,718
Satisfaction	FX	OA	0,930
Survival analisys	FX	ANK	0,406
Survival analisys	FX	RA	0,099
Survival analisys	FX	INS	0,095
Survival analisys	FX	OA	**0**,**047**

Long-term ulnar nerve disorders and QuickDASH, showed a worse outcome in the RA group (mean QuickDASH score: RA 46.4; OA 36.1; INS 31.4; FX 30.1; ANK 25.8) (ulnar nerve lesion: RA 1.4%; FX 1.1%; OA 0.9%; ANK 0.6%; INS 0.4%).

Again, when analyzing the two parameters, no statistically significant results emerged when comparing the various groups, as shown in Table 2.

Pain assessment using VAS gave very satisfactory cross-sectional results (a mean score of less than two out of 10 in all groups).

Long-term ulnar nerve disorders are more pronounced in the RA group, with the development of mild paresthesia in most cases (RA 1.4%; FX 1.1%; OA 0.9%; ANK 0.6%; INS 0.4%).

The evaluation of the subjective satisfaction of the patients on the result obtained from the surgical procedure is similar in the various groups (FX e INS 4.1; OA 4; RA 3.7; ANK 3.6).

The mean age of patients at the time of implantation is higher in the FX group; at the same time, this group has the shortest average clinical follow-up.

### Complications and failures

From the analysis of the medical records and from the postoperative clinical follow-up, adverse events and possible failures were collected among the 122 total elbow replacement implants reviewed.

In 27 instances (22.1%), there was reported involvement of the ulnar nerve to varying degrees. Among these, 20 cases (16.4%) manifested as more or less evident paresthesia, while seven cases (5.7%) exhibited a of the hypothenar eminence hypotrophy. Notably, two instances (1.6%) involved the development of transient paresthesia in the radial nerve, with subsequent complete recovery.

In one case (0.8%) triceps insufficiency was reported resulting in difficulty in executing an elbow extension against gravity.

In one additional single case there was a development of exuberant peri-implant heterotopic ossifications that limited the patient’s joint recovery.

**Fig. 2:**
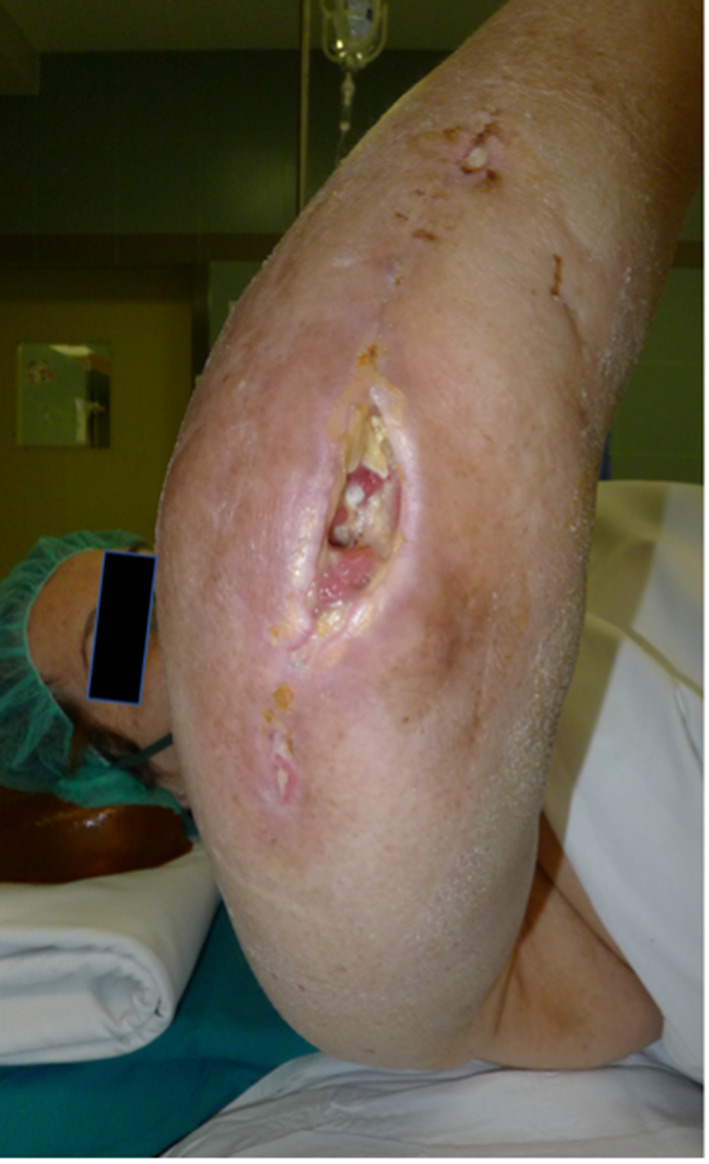
Dehiscience of surgical wound and acute periprosthetic infection

In three prostheses (2.5%) there was a dehiscience of the surgical wound and a difficult healing of the same, however without the subsequent development of an acute periprosthetic infection (Fig. 2).

**Fig. 3:**
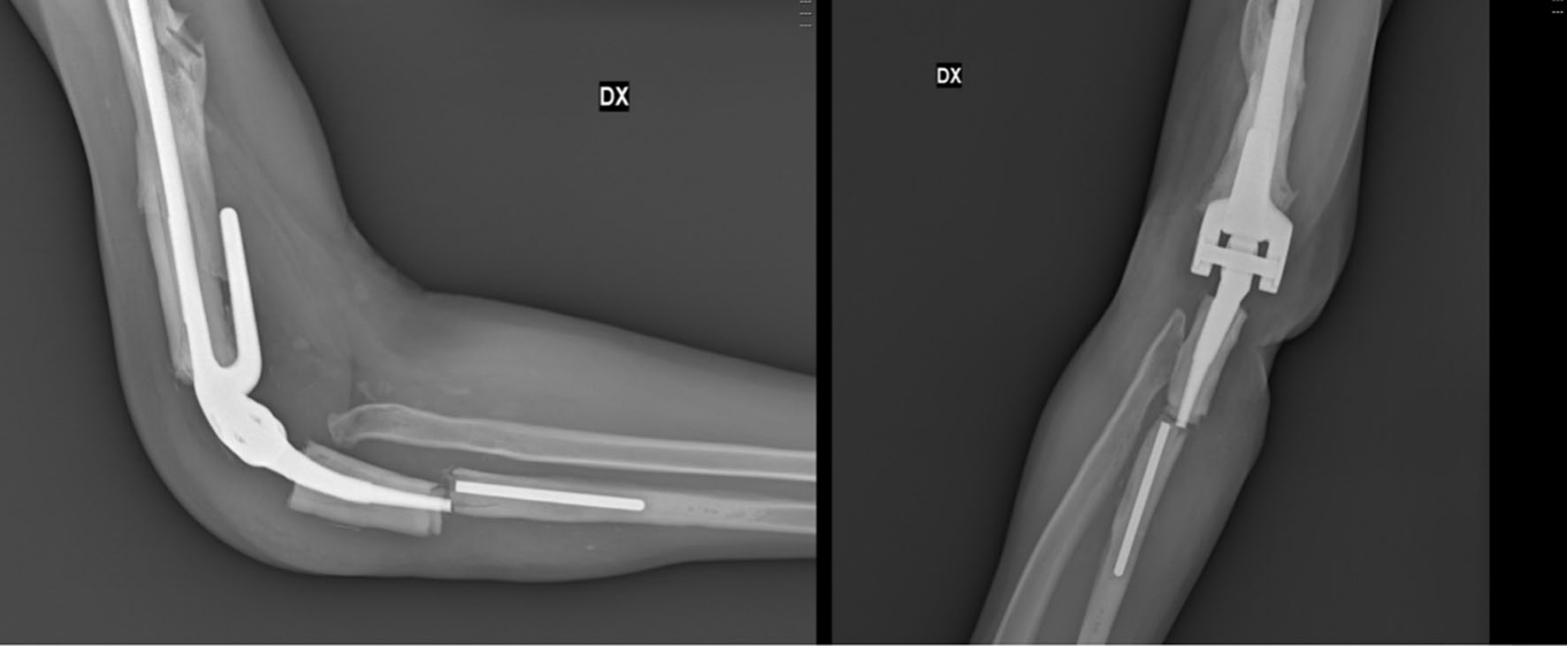
X-rays of a periprosthetic ulnar fracture and Coonrad Morrey stem fracture

**Fig. 4:**
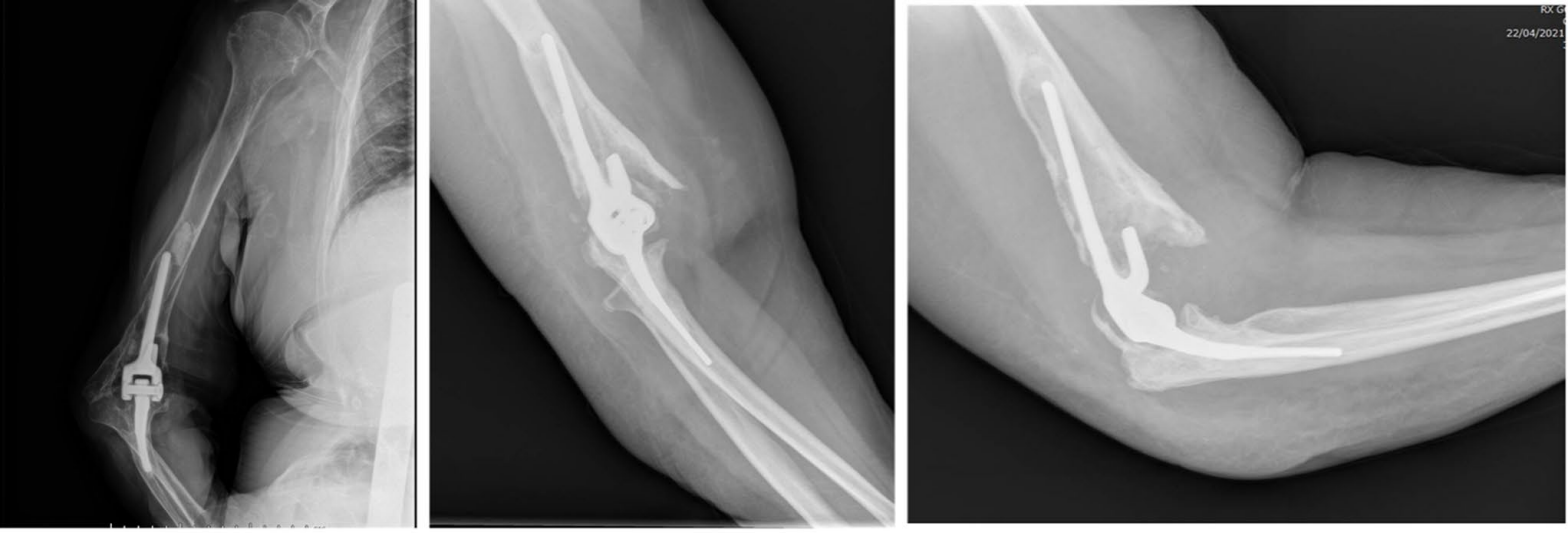
X-rays of aseptic loosening

**Fig. 5:**
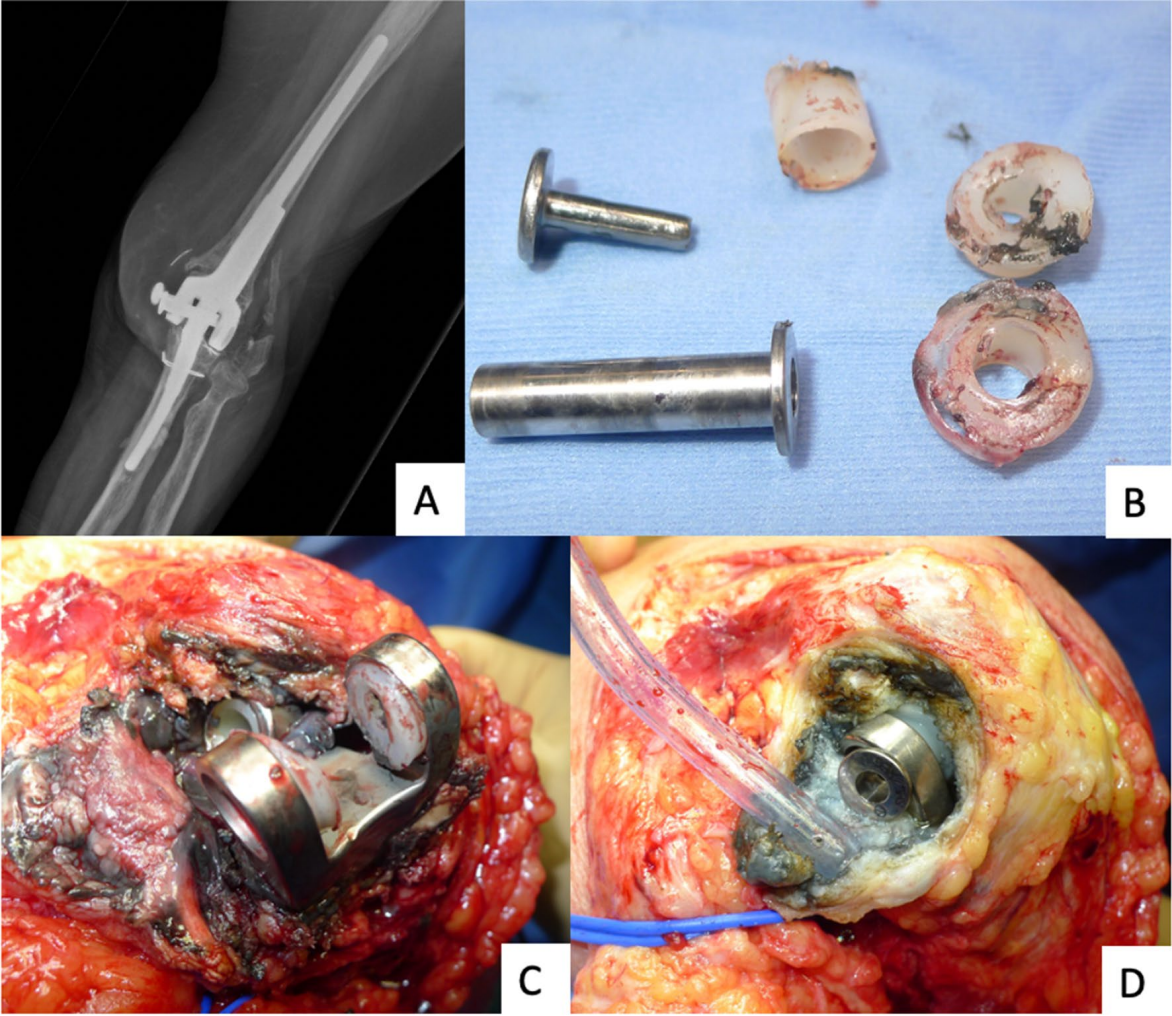
(**A**) X-ray of pin loosening after wearing of bushing’s polyethylene component; (**B**) (**C**) (**D**) intraoperative images of pin loosening after asymmetrical wearing of bushing’s polyethylene component

Twelve (9.8%) failures were reported among the prosthetic implants placed and for which revision surgery was subsequently required: seven (5.7%) aseptic loosening of the implant, of which one was following a periprosthetic fracture (Fig. 3), four (3.3%) late aseptic loosening (more than 12 months after primary surgery) (Fig. 4), and one case (0.8%) of wear of the polyethylene component of the bushing (Fig. 5), for which only a replacement of the worn component was necessary.

The survival time of CM arthroplasty observed in our study is 90% at 5years (60 months), 85% at 10years (120 months) and 83% at 15years (180 months) (Graphic 2).

**Graphic 1:**
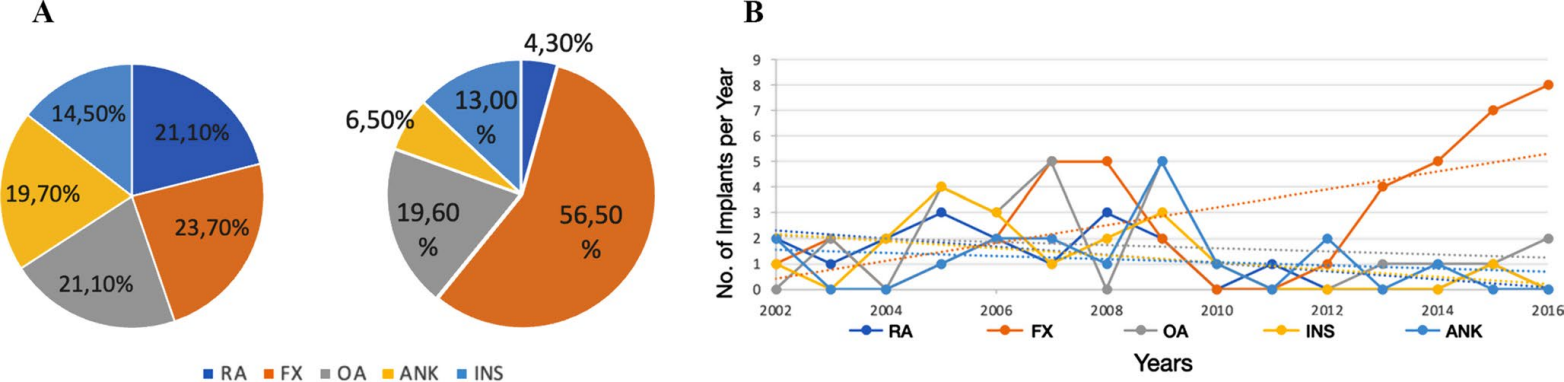
(**A**) Distributions compared in Interval A from October 2002 to September 2009 (on the left; substantial uniformity between the main applications of the prosthesis) and in Interval B from October 2009 to September 2016 (on the right; clear superiority of the TR group). (**B**) With each passing year there is an increase in the trend (expressed by the dotted lines) of the implantation of CM elbow prosthesis on fractures of the distal humerus

**Graphic 2:**
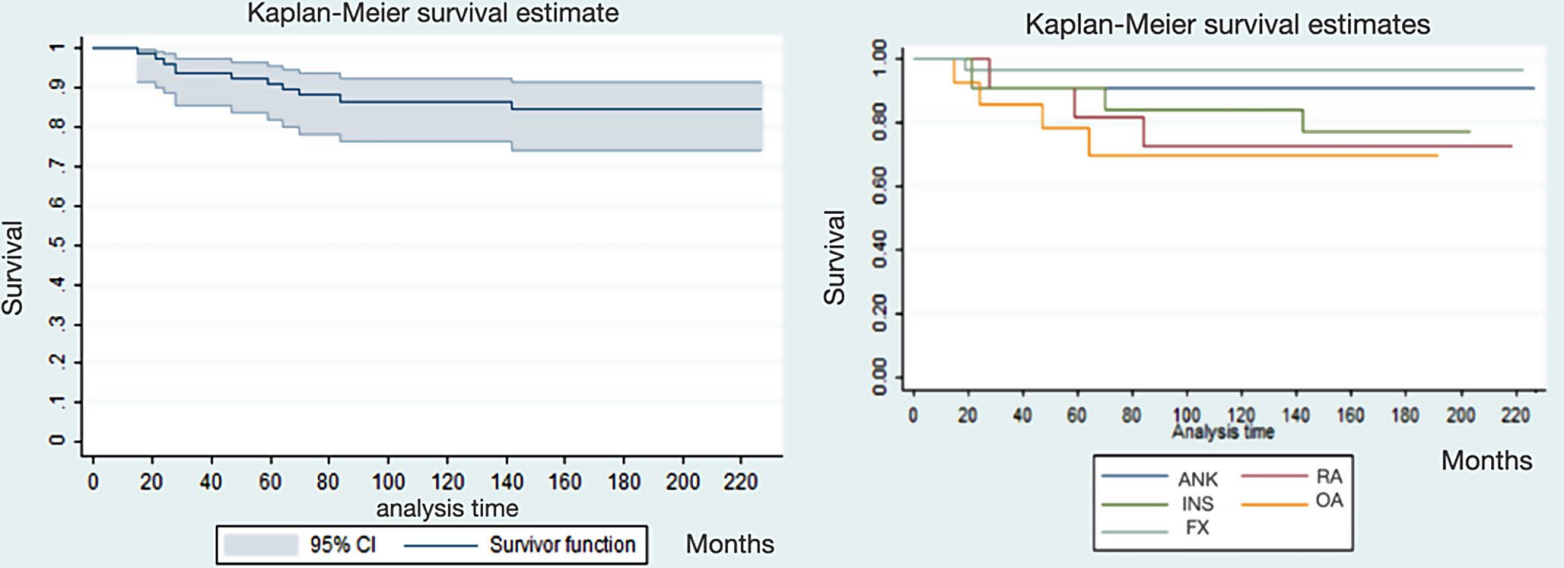
Kaplan-Meier survival function of CM implants: survival time of 90% at 60 months (5years), 85% at 120 months (10 years) and 83% at 180 months (15 years); the 95% confidence interval is indicated in light blue. Diversified survival function for the different indications that led to the prosthetic implant at 60 months (5 years) the survival rate was 97% for FX, 91% for INS and ANK, 82% for RA and 79% for OA while at 120 months (10 years) was 97% for FX, 91% for ANK, 84% for INS, 73% for RA and 70% for OA

The mean survival time of implants that failed was 50.1 ± 36.9 months (range 15 to 142), with our study showing that there were no statistically significant differences between groups in this parameter; except when survival time of the FX, with the best survival time, was compared to the OA, where we obtained a statistically significant result (p-value 0,047). The mean age of patients whose implants failed was 54.6 ± 15.1 years.

The cumulative Kaplan-Meier survival curve of this study identifies implant failure and subsequent revision surgery as the event determining the exit from the test group.

## Discussion

Before the 1990s, the main indication for elbow prosthesis was rheumatoid arthritis [4, 15–19], an indication which has undergone a progressive reduction especially in the last decade [18, 20–22].

Young et al [20]. in 2018 studied the correlation in RA patients between the use of DMARDs (“disease modifying antirheumatic drugs”) [20, 23, 24] and the reduction in the number of patients undergoing arthroplasty surgery in any joint district; the study highlighted a real decline in the number of elbow arthroplasty implants, greater than in any other joint district (from 24% in 2002 to 12% in 2012).

The results we obtained are in line with what has been published, with a significant decrease (87.5%) in cases of RA patients treated with CM prostheses: this may be correlated to the diffusion of these drugs has revolutionized the trend and the treatment of the pathology.

There has been a notable rise (44.4%) in the utilization of CM prostheses for the management of complex acute fractures of the distal humerus [25, 26].

This finding aligns with a recent systematic review conducted by Macken et al. [27]. in 2020.

This shift is mainly attributed to the increasing surgical confidence in applying a well-established technique. Additionally, elbow surgeons are increasingly seeking simpler and earlier patient rehabilitation without compromising the extensor mechanism, resulting in more reproducible outcomes compared to synthesis surgery, which can be challenging, particularly in the elderly, osteoporotic, and less compliant patients [27, 28].

Although several studies have analyzed the results of CM prostheses [8, 15, 16, 29–31], there is no systematic comparison of long-term results based on individual implant indications in the literature.

Revision surgery-free survival in our study was 90% at 5years (60 months), 85% at 10years (120 months) and 83% at 15years (180months); this finding is in line with the results of other studies in the literature [31, 32].

Only recently has a comparison been initiated between the implant indication and its longevity, revealing a generally higher failure rate in the AR group compared to FX group [21, 31, 32].

This study’s analysis included a comparison of patients with significant elbow instability, osteoarthritis, or ankylosis. The FX group exhibited a lower failure rate compared to the OA group, a finding corroborated by previous studies [21, 33], as well as INS, RA, and ANK groups. A high survival of the implant can therefore be more easily achieved in the acute treatment of a complex fracture of the distal humerus rather than in the subsequent execution of a prosthetic implant on a stiff, unstable or arthritic elbow (possible complications or outcomes of a previous trauma treated in another way); the data could, however, be explained by the higher age (average of 74.4 versus 62.5years) and the lower average monitoring (average of 84.6 versus 138.7months) of FX group compared to the others.

The complications and causes leading to revision after CM prosthesis implantation, as documented in our study, align with findings from comparable analyses in terms of patient cohorts or duration of clinical monitoring. Damage to the ulnar nerve is an often underestimated eventuality (between 2 and 26%) which, although in the majority of cases presents with minor paraesthesia and rarely with an involvement of the motor component (5.7%), constitutes a relatively frequent complication that should be made explicit in the collection of informed consent [4, 34].

The occurrence of wear in the high molecular weight polyethylene (UHMWPE) component of the prosthesis bushing is rare but feasible. However, aseptic loosening remains the predominant cause of revision surgery, constituting 5.7%, consistent with the literature’s range of 5–15% [18, 32, 35].

Conversely, septic mobilization exhibits a higher incidence (3.3%) compared to other joint areas like the hip and knee (0.5-2%) [36]. This disparity may be attributed in part to a significant number of patients with rheumatoid arthritis and concurrent use of immunosuppressive therapy. It is no coincidence that the studies in the literature that estimate the highest rates of periprosthetic infection (up to 9%) [36, 37] predate the diffusion of DMARDs.

The literature is full of studies that evaluate the functional results of the CM prosthesis, but there is a lack of data in this aspect that compare the various indications for the implant.

Mansat et al. and Hildebrand et al. [29, 31]. have presented generally better results in the group of patients suffering from rheumatoid arthritis when compared with those treated for traumatic pathologies, either in acute or for chronic complications.

Our work stands out in considering separately the implants performed on FX, OA, INS, RA, and ANK: this substantial difference in method justifies the profound differences in our results.

Using the MEPS, superior outcomes were observed in the FX, INS, and OA groups compared to the RA and ANK groups, although the latter exhibited generally satisfactory evaluations. Similarly, the greatest amplitude in the range of motion was achieved by FX, INS and OA, while a more limited mobilization was detected in ANK and RA patients, leading overall to a restoration of the functional range of motion (30–130°, so as defined by Morrey) in 64.7% of patients. The brightest results in particular were obtained in the OA group (*p* < 0.005).

The functionality of this joint clearly depends not only on the restoration of the joint surfaces but also on the quality of the surrounding soft tissues: greater retraction and reduced elasticity of these, present in patients with greater preoperative joint stiffness such as those affected by RA and by definition in those of the ANK group, justify the poor results; on the contrary, in the presence of a pathological picture that affects the articular surfaces only (OA group) with greater selectivity, greater recovery can be achieved.

The QuickDASH assesses residual disability across the entire upper extremity. Conditions with potential multi-district involvement, such as rheumatoid arthritis and osteoarthritis, may consequently not result in complete restoration of limb function during examination. Notably, RA and OA exhibit the least favourable outcomes among the examined groups.

The pain assessment using the VAS scale led across the board to very satisfactory results: 72.5% did not report any painful symptoms and no patient complained of moderate-severe pain (VAS ≥ 6).

Finally, we deemed it valuable to assess the subjective satisfaction level of the patients. Despite the heterogeneity observed across the examined cohorts (with ages ranging from 39 to 88years) and diverse initial pathological conditions, the proposed surgical intervention consistently met the needs and expectations of the patients across the board.

## Limitations

The prolonged duration covered in a substantial patient cohort, often including elderly individuals, led to the inability to follow-up on a significant number of cases. Furthermore, the absence of preoperative data posed challenges in evaluating the improvement in elbow functionality resulting from the surgical procedure. The study’s statistical strength was impacted by the relatively small patient population and the subdivision into multiple cohorts, a necessity for conducting a comparative analysis among diverse indications for CM implantation.

## Conclusions

The Coonrad-Morrey prosthesis has shown solid, satisfactory clinical results in the treatment of a wide range of pathologies that destroy the elbow joint. The indication for this type of operation has changed considerably over the years: the change is mainly due to an ever-increasing diffusion of the surgical technique and the introduction of new therapeutic models in the treatment of rheumatoid arthritis.

The durability of the prostheses over the long-term was satisfactory, yet the occurrence of failures and complications cannot be overlooked. The emergence of paresthesia within the ulnar nerve territory stands out as a complication that is frequently underestimated in both incidence and significance, emphasizing the importance for surgeons to thoroughly inform patients about this potential outcome during the informed consent process.

The implant in an elbow affected by rheumatoid arthritis or severe stiffness seems to have inferior results: we believe that the continuation of data collection carried out on a larger cohort of patients could increase the statistical power of this analysis. However, there was a good recovery in quality of life, free from pain and with limited residual disability of the limb.

Implantation of a Coonrad-Morrey prosthesis remains a procedure that requires considerable experience and familiarity with elbow surgery, particularly in managing the complex scenarios of revision surgery. However, given the positive subjective feedback expressed by patients and the encouraging long-term results, we believe that all this should not be a reason to give up considering this surgical option, particularly in the acute treatment of a complex fracture of the distal humerus in the elderly.

## Data Availability

Dehghan N, Furey M, Schemitsch L, Ristevski B, Goetz T, Schemitsch EH, Canadian Orthopaedic Trauma Society (COTS), McKee M (2019) Long-term outcomes of total elbow arthroplasty for distal humeral fracture: results from a prior randomized clinical trial. J Shoulder Elb Surg 28(11):2198– 2204. 10.1016/j.jse.2019.06.004
